# Supplementary material for: Annotation of natural product compound families using molecular networking topology and structural similarity fingerprinting
Source: Nat Commun. 2023 Jan 19;14:308. doi: 10.1038/s41467-022-35734-z (PMC9852437; doi:10.1038/s41467-022-35734-z)
Supplement: Supplementary file 1 — Supplementary information [file 41467_2022_35734_MOESM1_ESM.pdf]

Supplementary Information

Annotation of natural product compound families using molecular networking topology and structural similarity fingerprinting

Nicholas J. Morehouse, Trevor N. Clark, Emily J. McMann, Jeffrey A. van Santen, Jake F. P. Haeckl, Christopher A. Gray and Roger G. Linington*

CONTENTS

Table 1. Glossary of terms relevant to the SNAP-MS platform.

Table 2. Summary of SNAP-MS results when analyzing a filtered version of the molecular network built using the NIH Natural Products Library Round 1 and Round 2 with the COCONUT database.

Table 3. Summary of Network Annotation Propagation results when analyzing a filtered version of the molecular network built using the NIH Natural Products Library Round 1 and Round 2 using the COCONUT database. This summary only looks at the results of consensus scoring.

Table 4. Parameters selected for SNAP-MS analysis of mass spectrometry datasets.

Figure 1. Distribution of formulae within the Natural Products Atlas. (a) full distribution of all formulae (b) expansion in the region containing 15 carbons highlighting the most frequently occurring formulae.

Figure 2. Distribution of molecular formula across compound families within the Natural Products Atlas, excluding compound families with two or fewer members.

Figure 3. Intra-family distributions of molecular formulae. The log-transformed occurrence (bar charts) or proportion (pie charts) of the number of distinct compound families containing (a,b) a single molecular formula, (c,d) a pair of molecular formulae or (e,f) a set of three molecular formulae. appearing in one or more compound families. This includes formulae that only occur once within the Natural Products Atlas.

Figure 4. Subnetworks from the NIH Natural Products Library datasets that were incorrectly identified by SNAP-MS.

Figure 5. Validation of the subnetwork from the actinobacterial extract library dataset identified to be desferrioxamines. (a) The SNAP-MS compound family identification with desferrioxamine E indicated by a square node. (b) Mass spectrum and (c) ^{1}H NMR of desferrioxamine E isolated from prefraction RLUS-2153C acquired at 600 MHz in CD_3OD.

Figure 6. Validation of the subnetwork from the actinobacterial extract library dataset identified to be surugamides. (a) The SNAP-MS compound family identification with surugamide A indicated by square nodes. (b) Mass spectrum and (c) ^{1}H NMR of surugamide A isolated from prefraction RLUS-2144D acquired at 600 MHz in DMSO-d_6.

Figure 7. Validation of the subnetwork from the actinobacterial extract library dataset identified to be mycosubtilins. (a) The SNAP-MS compound family identification with mycosubtilin D indicated by a square node. (b) Mass spectrum and (c) ^{1}H NMR of mycosubtilin D isolated from prefractions RLUS-2090B, C, and D acquired at 600 MHz in DMSO-d_6.

Figure 8. Validation of the subnetwork from the actinobacterial extract library dataset identified to be enterocins. (a) The top ranked SNAP-MS compound family identifications with enterocin indicated by a square node. (b) Comparison of mass spectra and (c) extracted

ion chromatograms of authentic enterocin, the prefraction RLUS-2153D and authentic enterocin in prefraction RLUS-2153D.

Figure 9. Validation of the subnetwork from the actinobacterial extract library dataset identified to be CDAs. (a) The SNAP-MS compound family identification with CDA3A indicated by a square node. (b) Comparison of mass spectra and (c) extracted ion chromatograms of authentic CDA, the prefraction RLUS-2052C and authentic CDA in prefraction RLUS-2052C.

Figure 10. Validation of the subnetwork from the actinobacterial extract library dataset identified to be amicoumacins. (a) The SNAP-MS compound family identification with AI-77-B indicated by a square node. (b) Comparison of MS^2 data of amicoumacin B with isolated AI-77-B. (c) The ^1H NMR of AI-77-B isolated from prefraction RLUS-2079C.

Figure 11. Validation of the subnetwork from the actinobacterial extract library dataset identified to be nactins. (a) The SNAP-MS compound family identification. (b) MS^1 of the isolated nactin analogue showing in source fragmentation. (c) The ^1H NMR of a nactin analogue isolated from prefraction RLUS-2210D acquired at 600 MHz in $CDCl_3$.

Figure 12. The SNAP-MS dashboard available via the Natural Products Atlas website (https://www.npatlas.org/discover/snapms/).

Figure 13. The SNAP-MS results page.

Figure 14. Additional compound family prediction made by analyzing the actinobacterial derived molecular network with SNAP-MS filtered to search bacterial natural products.

Note 1. In house bacterial extract library creation and data acquisition for molecular networking.

Note 2. SNAP-MS validation.

Note 3. Comparison of annotation accuracy between SNAP-MS and Network Annotation Propagation (NAP).

Note 4. Cosine scoring with *in silico* MS^2 prediction

Figure 15. Cosine scoring of NIH DDA data to CFM-ID *in silico* MS^2 data. Examples of using cosine scoring to rank SNAP-MS results for three different GNPS subnetworks (A, B, and C). Cosine score for selected (yellow) nodes shown. The black box is used to designate the correct answer. Nodes with dark blue borders show SNAP-MS top hits.

Figure 16. Cosine scoring of marine actinomycete extract DIA data against CFM-ID *in silico* MS^2 data. Examples of using cosine scoring to rank SNAP-MS results for six different GNPS subnetworks (A-F). Cosine score for selected nodes in A-F shown by color scheme G with light gray being features that were not M+H and were not compared, and yellow being a score of 0. The black box is used to designate the correct answer. Nodes with dark green borders show SNAP-MS top hits.

Supplementary Table 1 | Glossary of terms relevant to the SNAP-MS platform.

Context	Term	Definition
SNAP-MS	Compound family	Discreet groupings of molecules from a compound database, generated using structural similarity. Compound family constitution can vary depending on the selected chemical fingerprinting method, similarity score threshold, and chemical database.
SNAP-MS	Structural similarity network	A graphical representation of the relatedness of molecules (grouped into compound families) based on their structural features.
SNAP-MS	Chemical fingerprint	A numerical representation of a molecule that allows for a quantifiable comparison of the similarity of two molecules.
GNPS[1]	Molecular Network	A graphical representation of tandem mass spectrometry experiments that groups similar fragmentation spectra.
GNPS	Subnetwork (also molecular family or spectral family)	A group of interconnected nodes corresponding to similar fragmentation spectra.
GNPS	Component index	Unique, numerical identifiers for subnetworks in a molecular network.
GNPS	MS^2 spectral networking	The grouping of MS-2 spectra into a molecular network
GNPS	MS^2 spectral matching	The comparison of MS-2 spectra of unknown metabolites with reference spectra.
CANOPUS[2]	Compound classes	A subset of the ChemOnt ontology of ClassyFire consisting of 2,497 classes.

Supplementary Table 2 | Summary of SNAP-MS results when analyzing a filtered version of the molecular network built using the NIH Natural Products Library Round 1 and Round 2 with the COCONUT database.

	True Positive	False Positive	True Negative	False Negative
Expected	278	0	16	0
Observed	164	20	16	94

True Positive: When the top ranked result or one of the tied top ranked results matches the compound family from the original subnetwork. We expect true positives for subnetworks where three or more molecules with unique molecular formulae are also found in the COCONUT database.

False Positive: Any time the correct answer is not amongst the top ranked answer when it was expected to be.

True Negative: When SNAP-MS has not returned an answer when we expected no answer to be returned. We expect no answer in cases where there are only two or fewer COCONUT molecules with unique molecular formulae in a subnetwork.

False Negative: Any time SNAP-MS does not return an answer when a correct answer was expected.

Supplementary Table 3 | Summary of Network Annotation Propagation results when analyzing a filtered version of the molecular network built using the NIH Natural Products Library Round 1 and Round 2 using the COCONUT database. This summary only looks at the results of consensus scoring.

	True Positive	False Positive	True Negative	False Negative
Expected (≥33% coverage)	288	0	6	0
Expected (≥50% coverage)	284	0	10	0
Top ten annotations ≥33% coverage	229	63	1	1
Top ten annotations ≥50% coverage	211	81	1	1
Consensus score ≥0.9 ≥33% coverage	203	89	1	1
Consensus score ≥0.9 ≥50% coverage	183	109	1	1

True Positive: When NAP has identified the correct answer within the top ten candidate structures with a consensus score > 0 or ≥0.9 in ≥33 or ≥50% of nodes in a subnetwork. We expect true positives for subnetworks where at least ≥33% or ≥50% of the nodes are capable of being correctly identified.

False Positive: Any time less than ≥33% or ≥50% of the nodes in a subnetwork are correctly annotated.

True Negative: When NAP has not returned an answer when no answer was expected. We would expect no answer when <33% or <50% of the nodes are capable of being correctly identified.

False Negative: Any time NAP does not return an answer when a correct answer was expected.

Supplementary Table 4 | Parameters selected for SNAP-MS analysis of mass spectrometry datasets.

Dataset	Reference Database	Selected adducts	Ppm error	Minimum subnetwork size	Maximum subnetwork size	Minimum compound family size	Maximum results edges	Maximum results nodes
NIH Natural Products Library Round 1 and 2	Full NP Atlas	$[M+H]^+$, $[M+Na]^+$, $[M+NH_4]^+$, and $[M+H-H_2O]^+$	5	4	100	3	10,000	2,000
NIH Natural Products Library Round 1 and 2	COCONUT	$[M+H]^+$, $[M+Na]^+$, $[M+NH_4]^+$, and $[M+H-H_2O]^+$	5	4	100	3	10,000	2,000
Actinobacterial extract library	firmicutes and actinobacteria	$[M+H]^+$, $[M+Na]^+$, and $[M+H-H_2O]^+$	15	4	100	3	10,000	2,000
Tobias et al.[3]	Bacteria	$[M+H]^+$, $[M+Na]^+$, and $[M+H-H_2O]^+$	20	4	100	3	10,000	2,000
Nguyen et al.[4]	Bacteria	$[M+H]^+$, $[M+Na]^+$, and $[M+H-H_2O]^+$	40	4	100	3	10,000	2,000
Mudalungu et al.[5]	Bacteria	$[M+H]^+$, $[M+Na]^+$, and $[M+H-H_2O]^+$	20	4	100	3	10,000	2,000
Caraballo-Rodríguez, Dorrestein and Pupo[6]	Full NP Atlas	$[M+H]^+$, $[M+Na]^+$, and $[M+H-H_2O]^+$	20	4	100	3	10,000	2,000

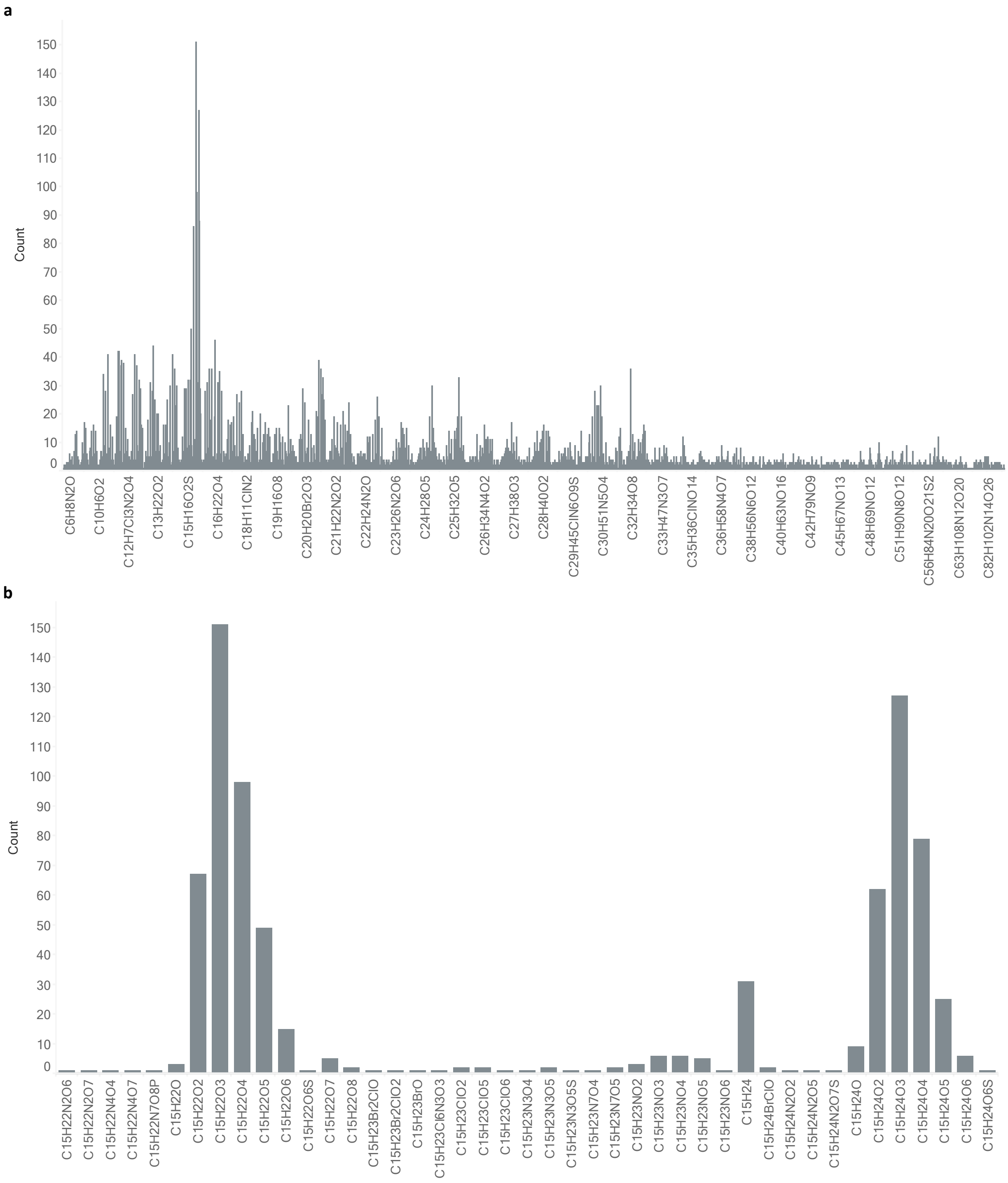

Supplementary Figure 1 | Distribution of formulae within the Natural Products Atlas. (a) full distribution of all formulae (b) expansion in the region containing 15 carbons highlighting the most frequently occurring formulae.

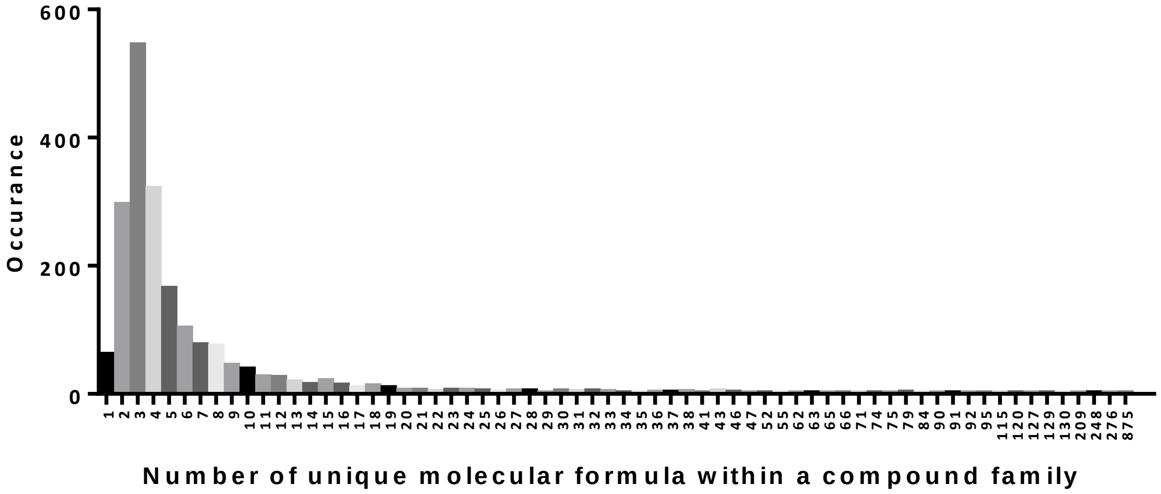

Supplementary Figure 2 | Distribution of molecular formula across compound families within the Natural Products Atlas, excluding compound families with two or fewer members.

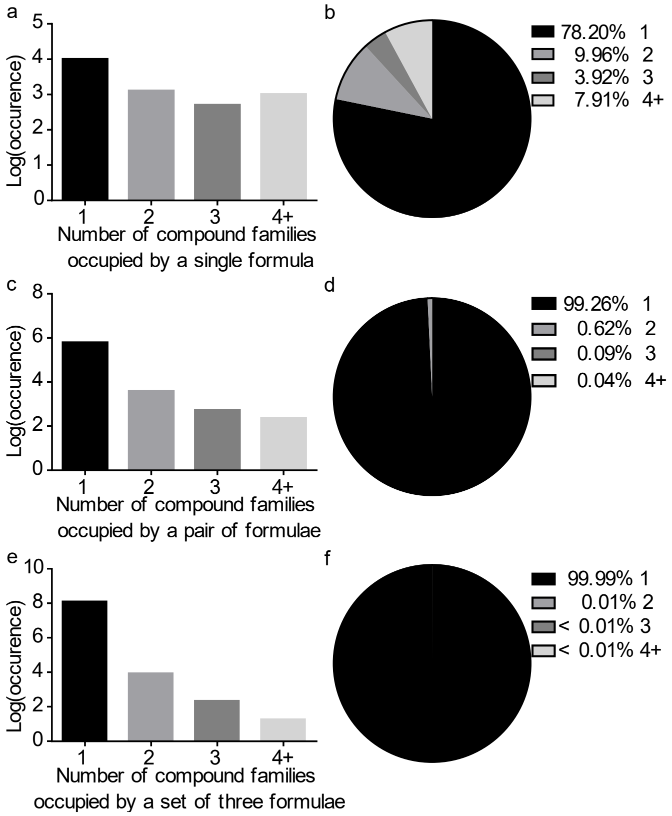

Supplementary Figure 3 |Intra-family distributions of molecular formulae. The log-transformed occurrence (bar charts) or proportion (pie charts) of the number of distinct compound families containing (a,b) a single molecular formula, (c,d) a pair of molecular formulae or (e,f) a set of three molecular formulae. appearing in one or more compound families. This includes formulae that only occur once within the Natural Products Atlas.

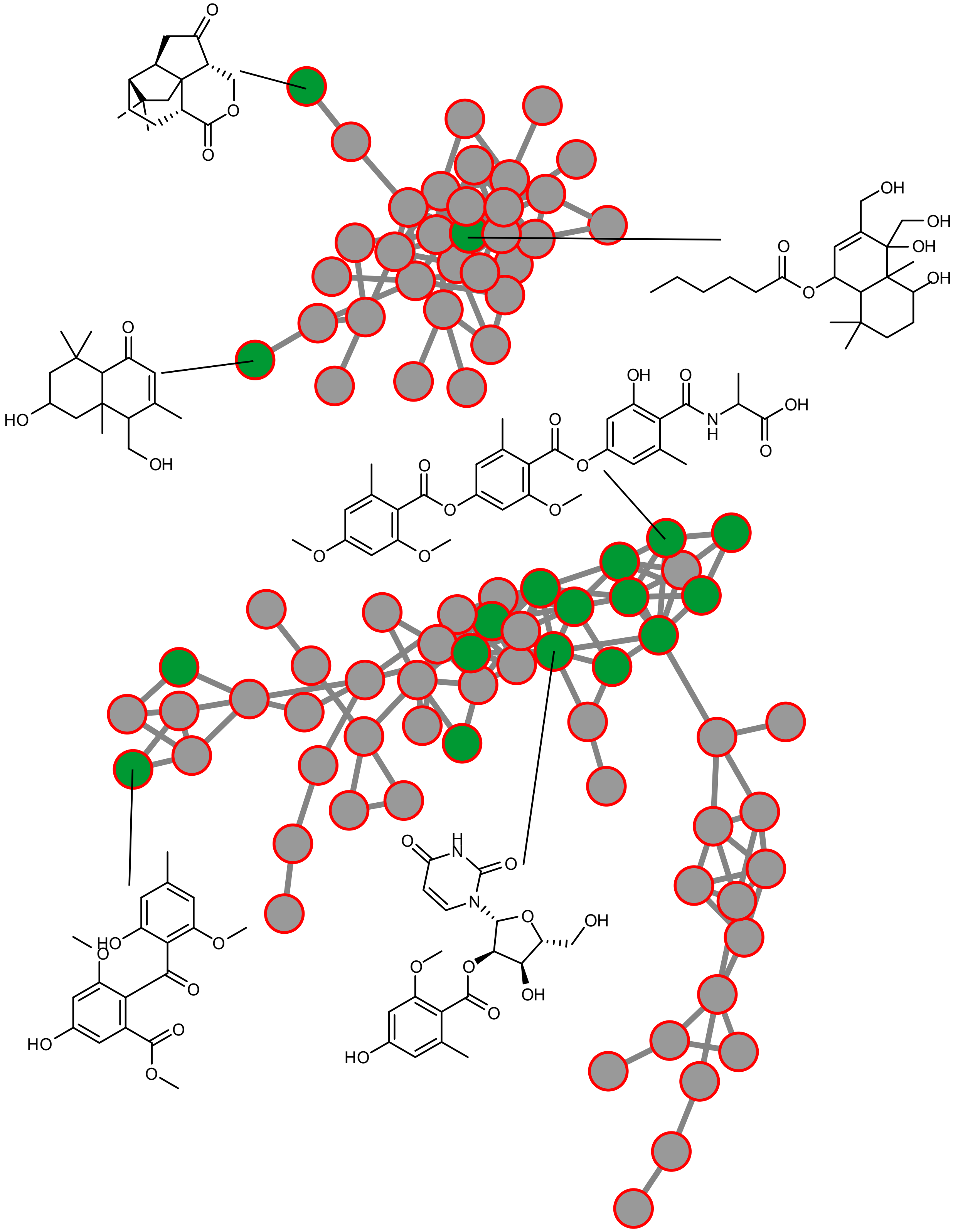

Supplementary Figure 4 | Subnetworks from the NIH Natural Products Library datasets that were incorrectly identified by SNAP-MS.

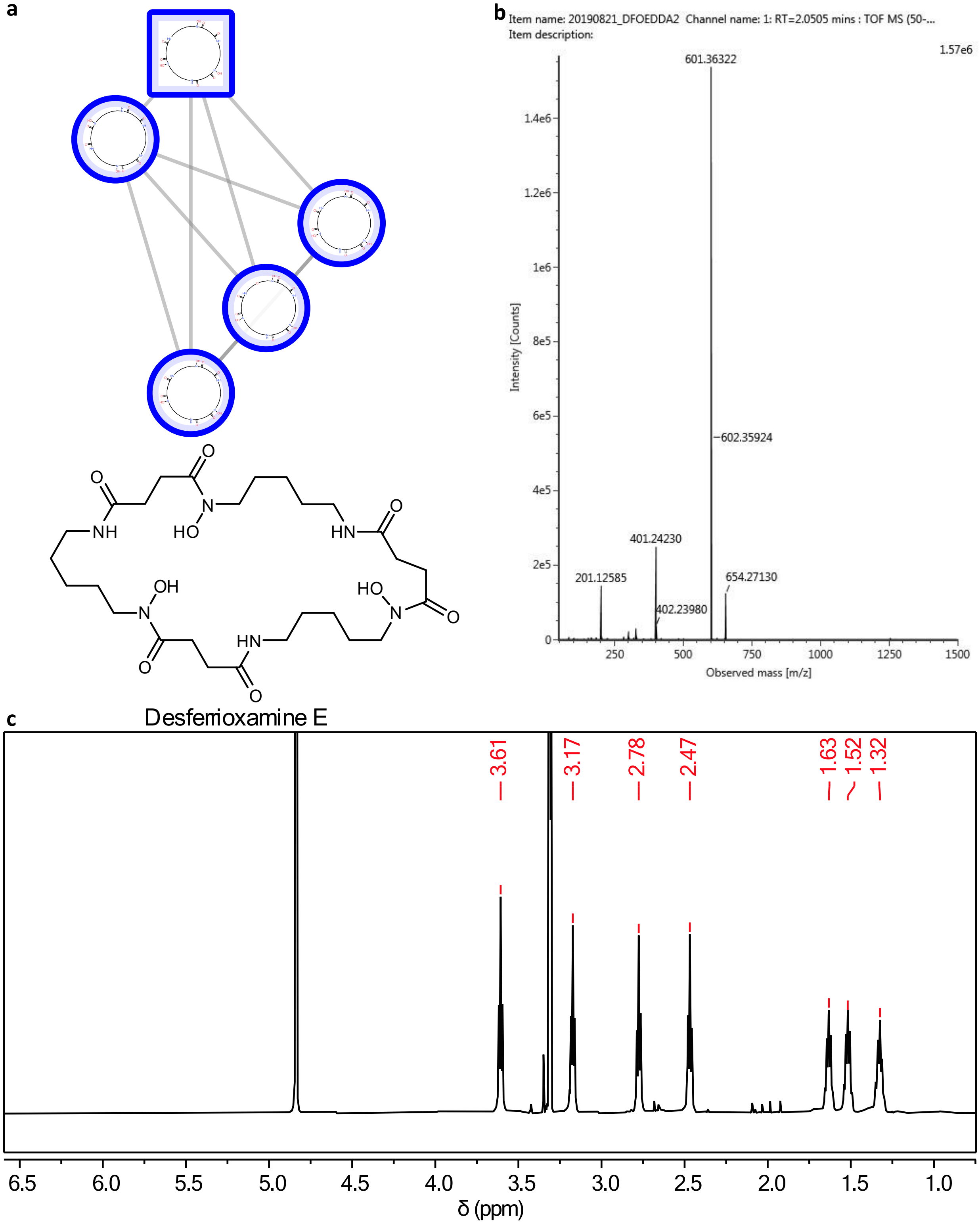

Supplementary Figure 5 | Validation of the subnetwork from the actinobacterial extract library dataset identified to be desferrioxamines. (a) The SNAP-MS compound family identification with desferrioxamine E indicated by a square node. (b) Mass spectrum and (c) ^{1}H NMR of desferrioxamine E isolated from prefraction RLUS-2153C acquired at 600 MHz in CD_3OD.

a

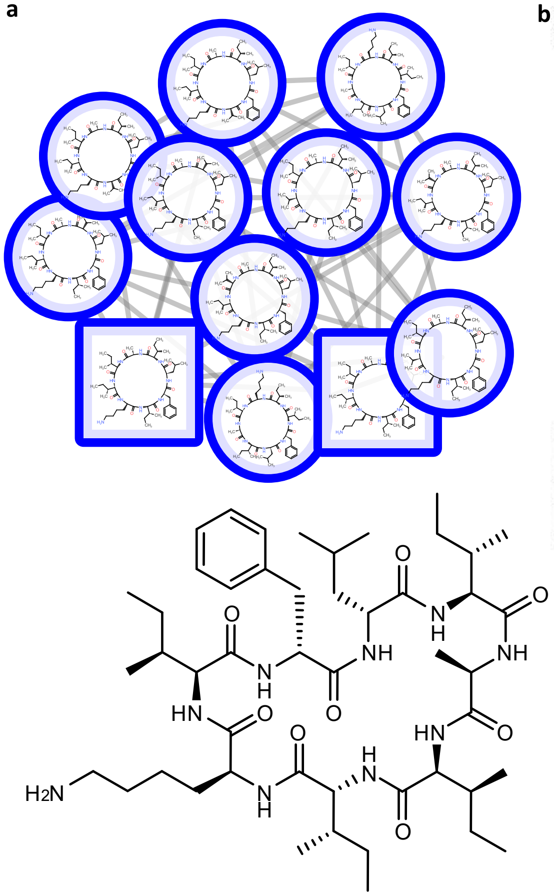

Surugamide A

b

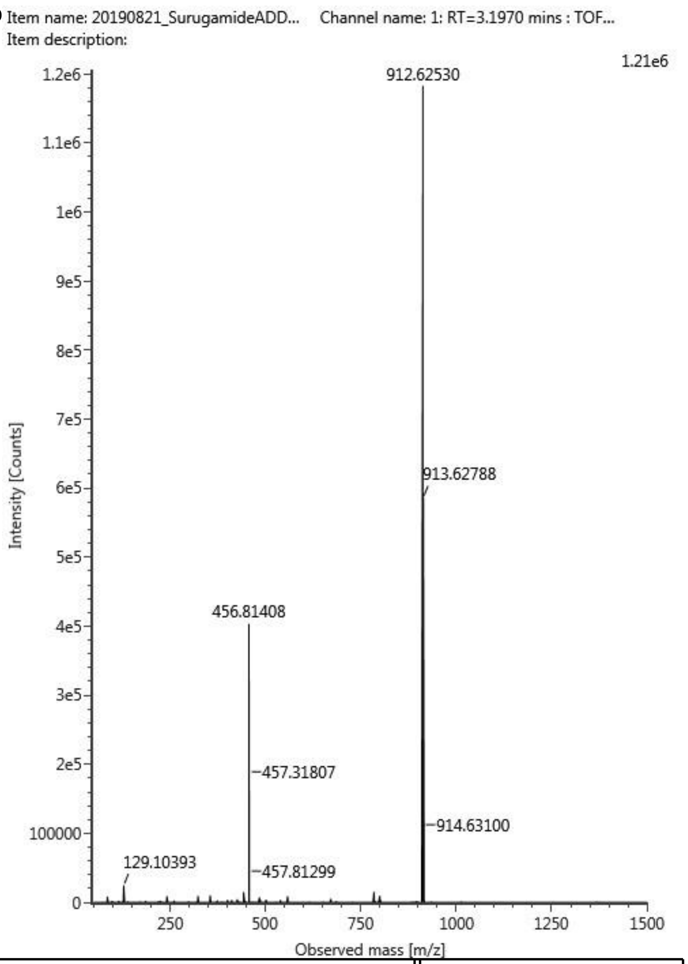

c

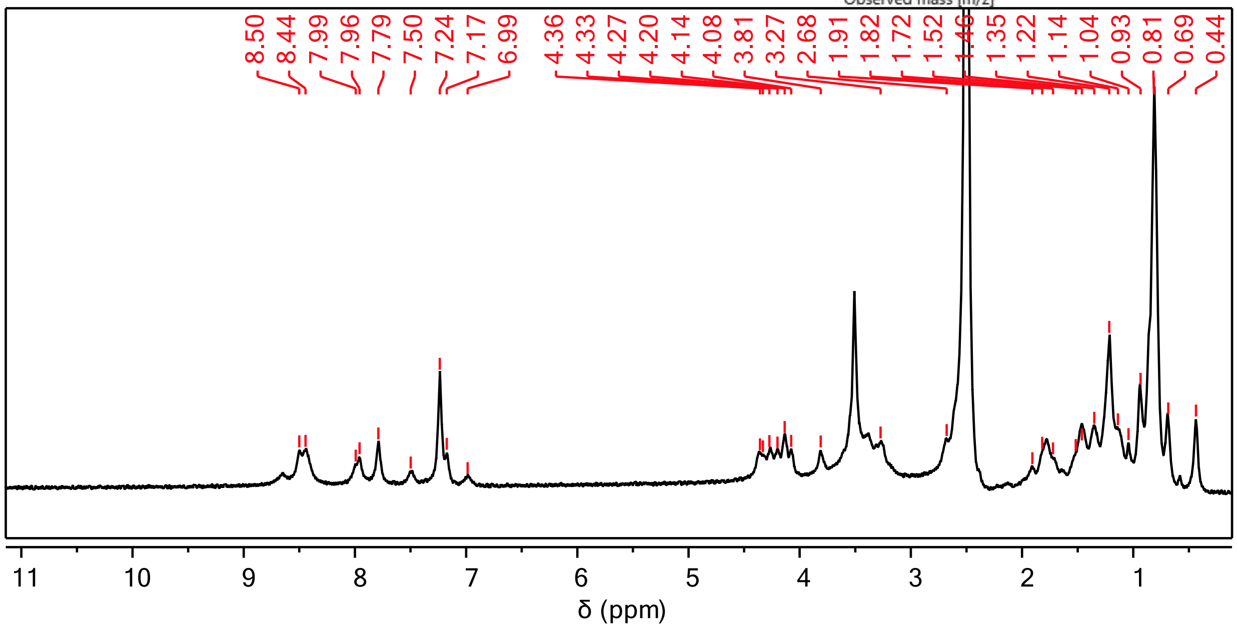

Supplementary Figure 6 | Validation of the subnetwork from the actinobacterial extract library dataset identified to be surugamides. (a) The SNAP-MS compound family identification with surugamide A indicated by square nodes. (b) Mass spectrum and (c) ^{1}H NMR of surugamide A isolated from prefraction RLUS-2144D acquired at 600 MHz in DMSO-d_6.

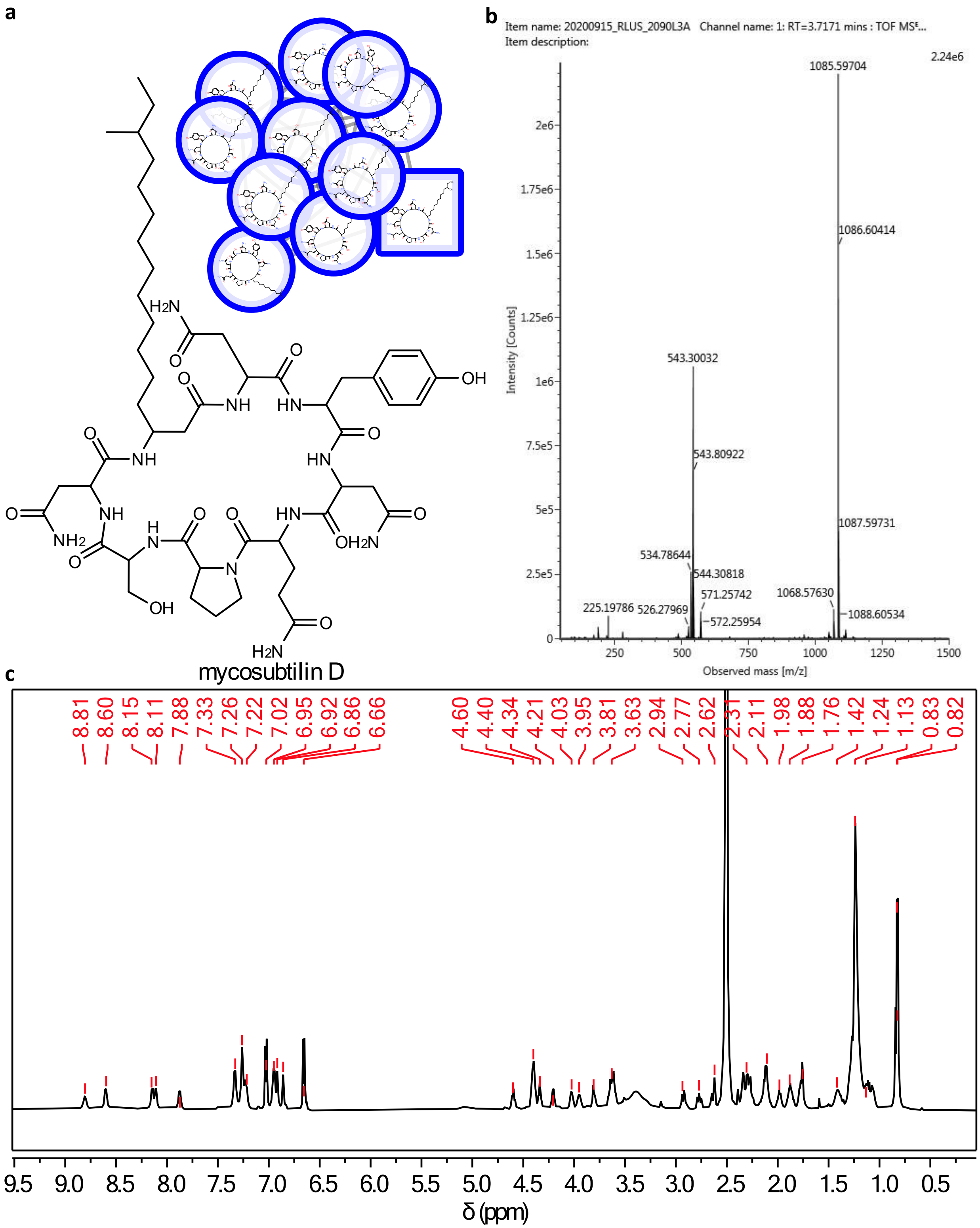

Supplementary Figure 7 | Validation of the subnetwork from the actinobacterial extract library dataset identified to be mycosubtilins. (a) The SNAP-MS compound family identification with mycosubtilin D indicated by a square node. (b) Mass spectrum and (c) ^{1}H NMR of mycosubtilin D isolated from prefractions RLUS-2090B, C, and D acquired at 600 MHz in DMSO-d_6.

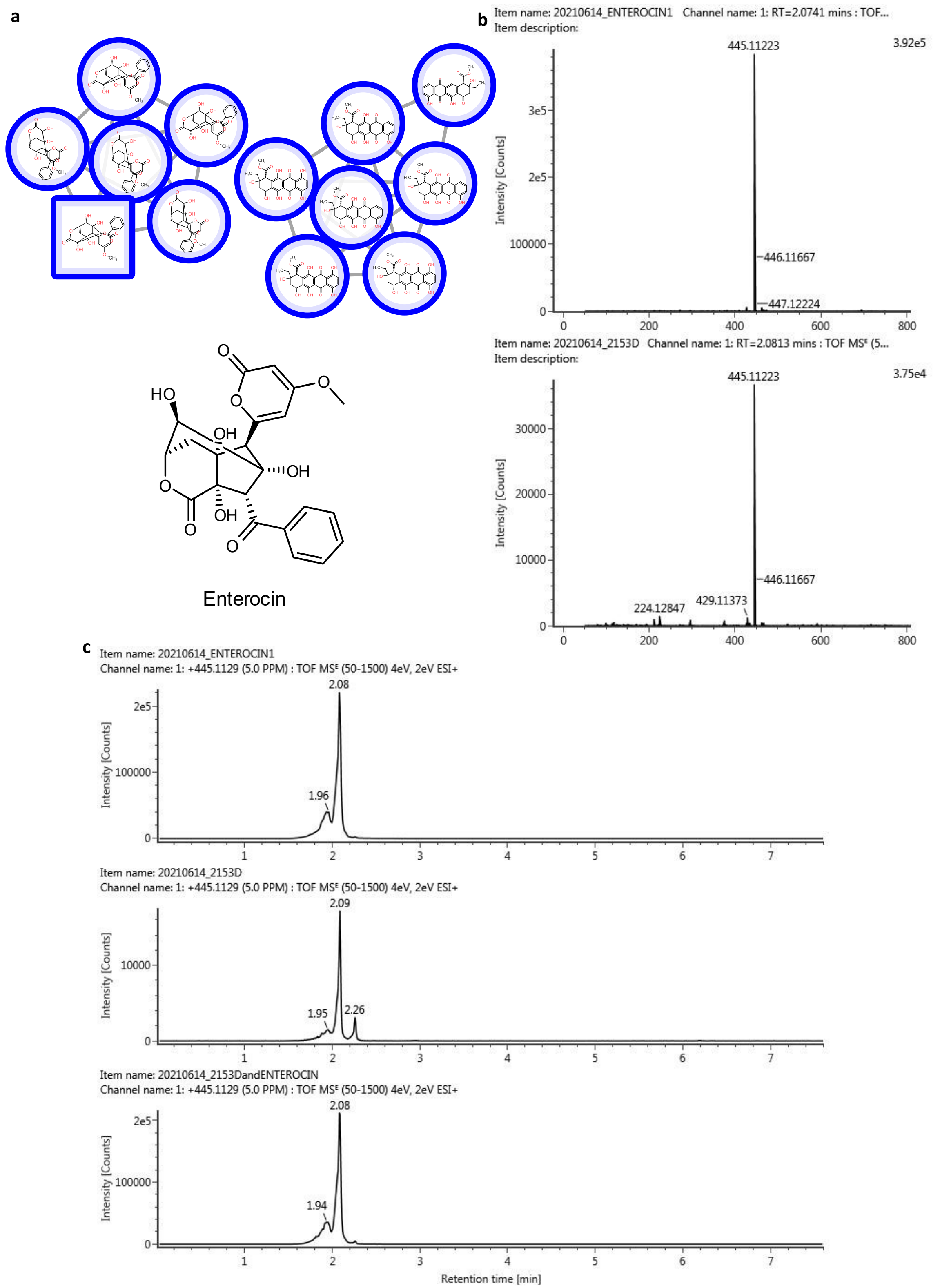

Supplementary Figure 8 | Validation of the subnetwork from the actinobacterial extract library dataset identified to be enterocins. (a) The top ranked SNAP-MS compound family identifications with enterocin indicated by a square node. (b) Comparison of mass spectra and (c) extracted ion chromatograms of authentic enterocin, the prefraction RLUS-2153D and authentic enterocin in prefraction RLUS-2153D.

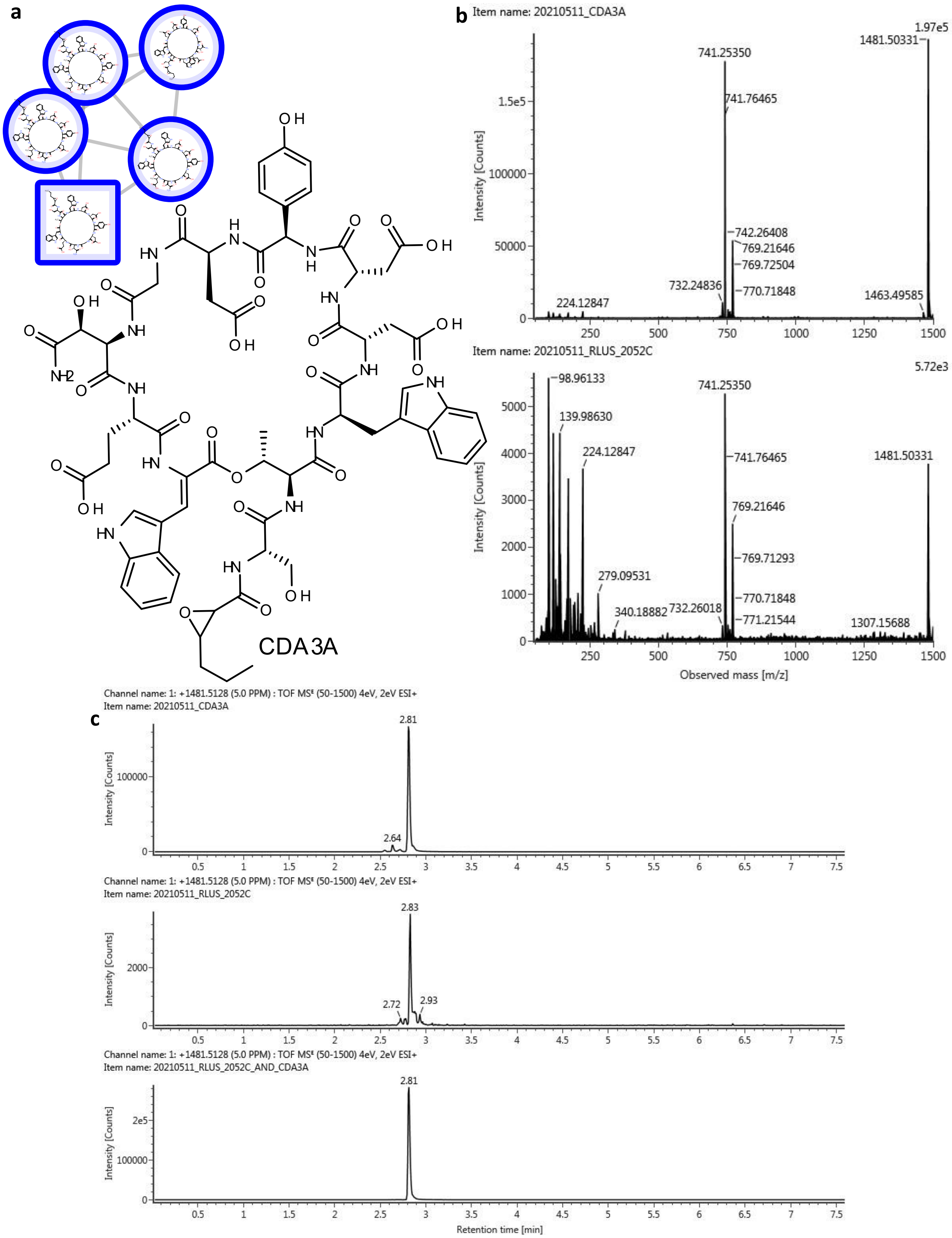

Supplementary Figure 9 | Validation of the subnetwork from the actinobacterial extract library dataset identified to be CDAs. (a) The SNAP-MS compound family identification with CDA3A indicated by a square node. (b) Comparison of mass spectra and (c) extracted ion chromatograms of authentic CDA, the prefraction RLUS-2052C and authentic CDA in prefraction RLUS-2052C.

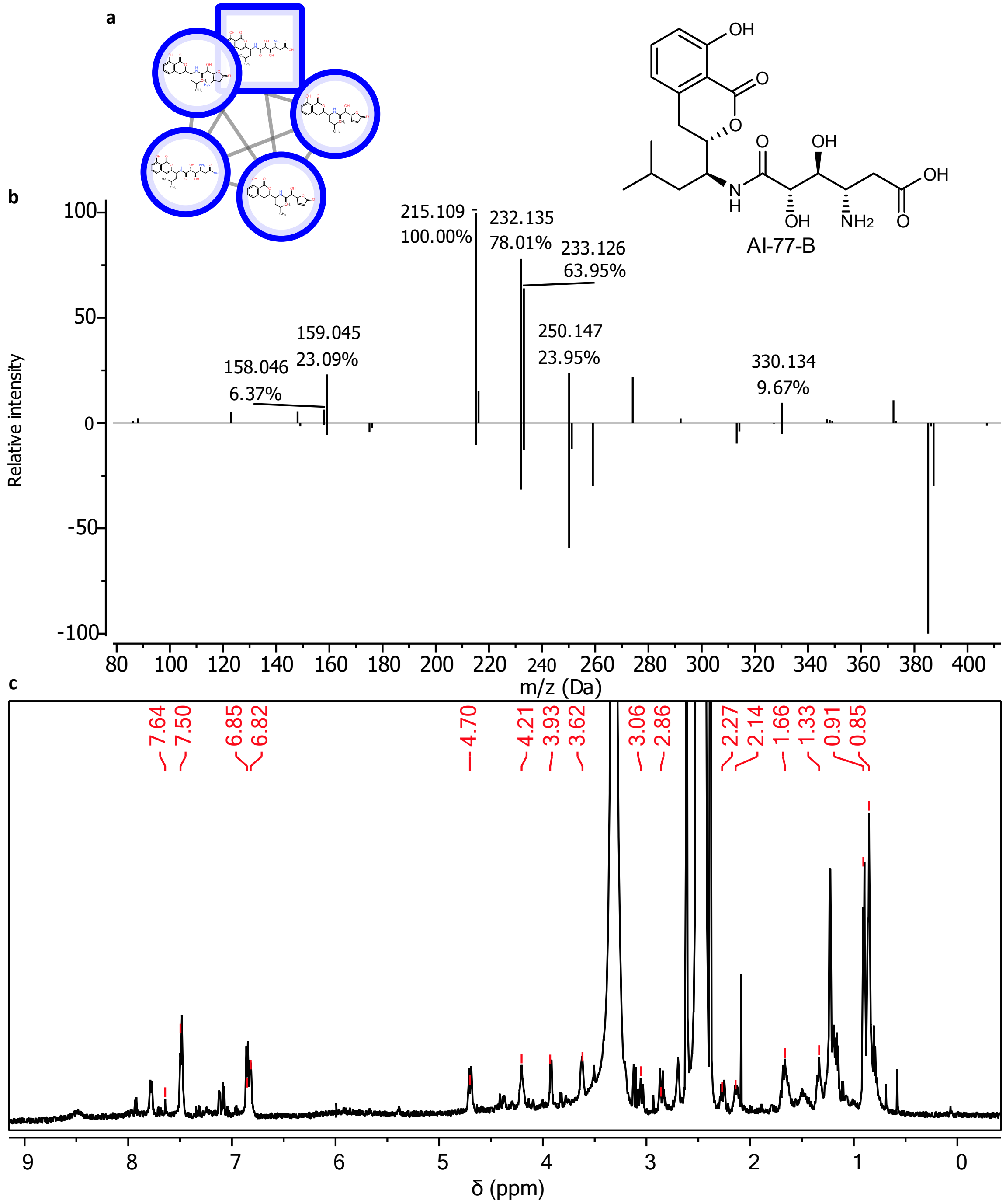

Supplementary Figure 10 | Validation of the subnetwork from the actinobacterial extract library dataset identified to be amicoumacins. (a) The SNAP-MS compound family identification with AI-77-B indicated by a square node. (b) Comparison of MS^2 data of amicoumacin B with isolated AI-77-B. (c) The ^1H NMR of AI-77-B isolated from prefraction RLUS-2079C.

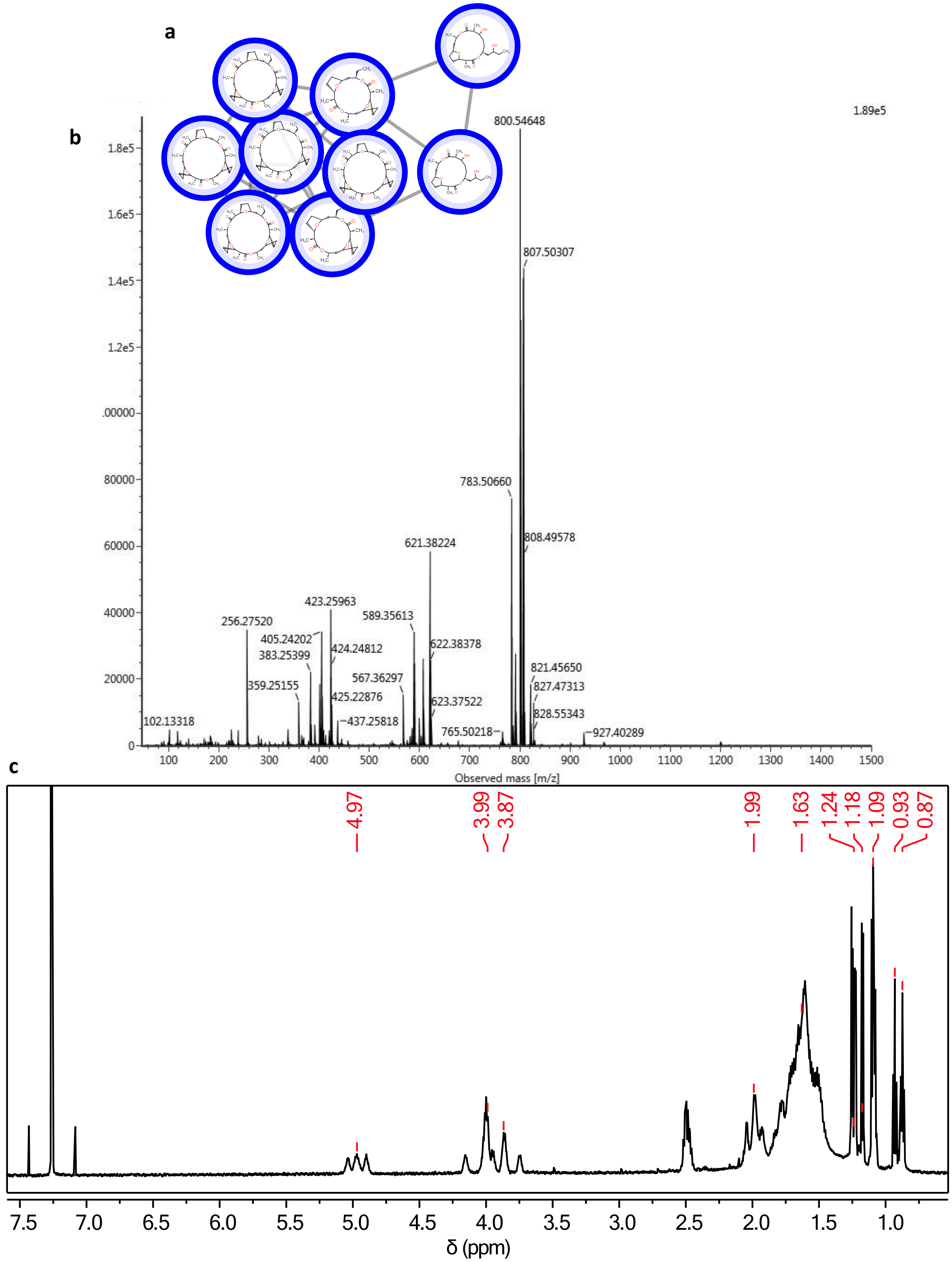

Supplementary Figure 11 | Validation of the subnetwork from the actinobacterial extract library dataset identified to be nactins. (a) The SNAP-MS compound family identification. (b) MS[1] of the isolated nactin analogue showing in source fragmentation. (c) The ^{1}H NMR of a nactin analogue isolated from prefraction RLUS-2210D acquired at 600 MHz in $CDCl_3$.

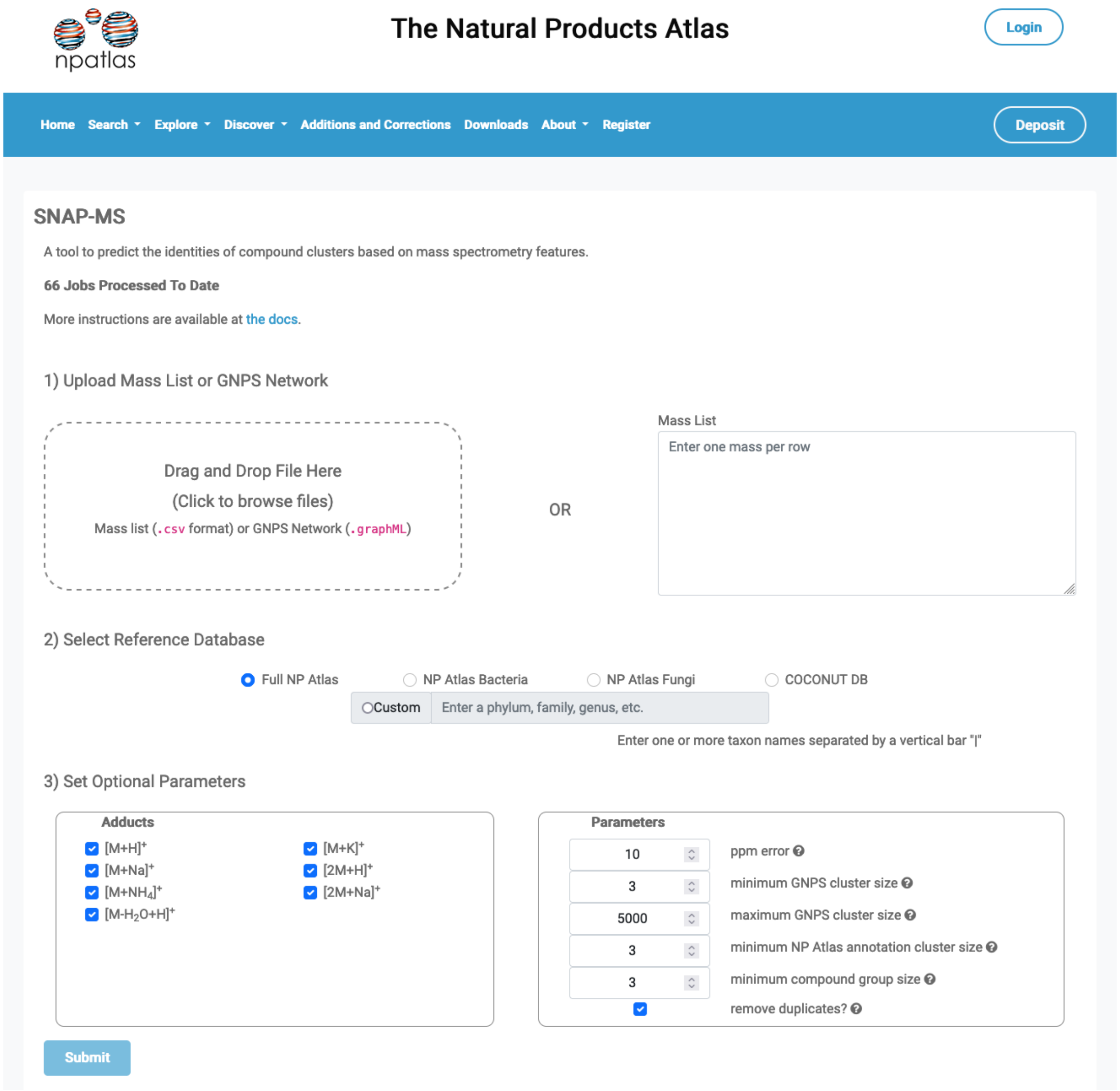

Supplementary Figure 12 | The SNAP-MS dashboard available via the Natural Products Atlas website (https://www.npatlas.org/discover/snapms/).

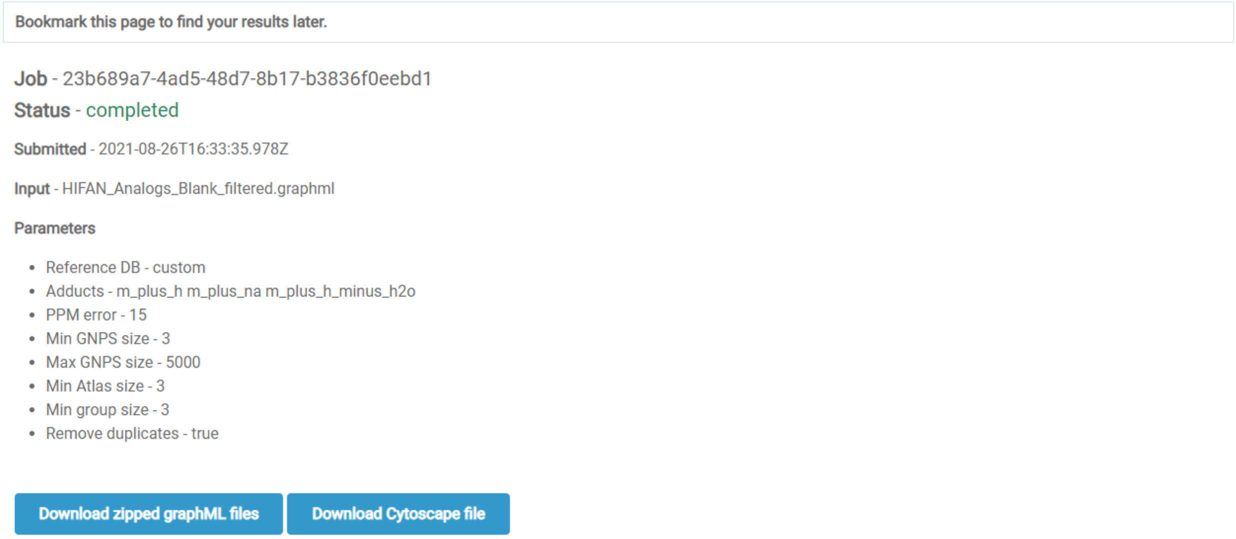

Supplementary Figure 13 | The SNAP-MS results page.

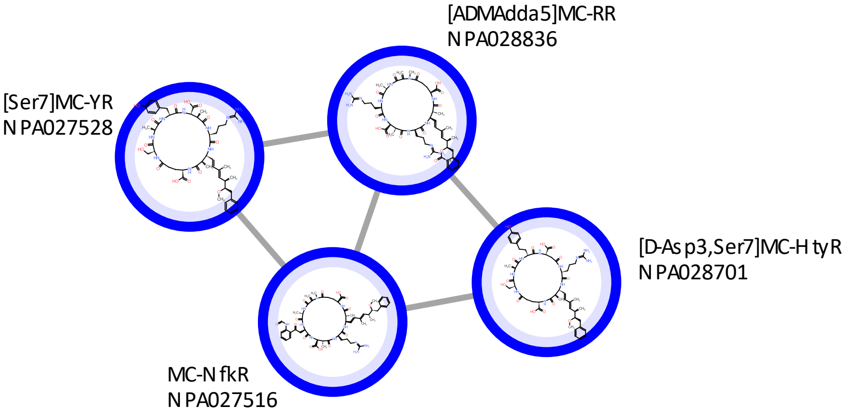

Supplementary Figure 14 | Additional compound family prediction made by analyzing the actinobacterial derived molecular network with SNAP-MS filtered to search bacterial natural products.

Supplementary Note 1. In house bacterial extract library creation and data acquisition for molecular networking.

Actinobacteria isolation and identification

Actinobacteria were isolated from marine sediment collected by SCUBA along the West coast of the United States, primarily around the San Juan Islands of Washington state. Samples were collected with 15-mL centrifuge tubes and plated or serially stamped onto six selection media agar plates for microbial isolation (AIS, AIF, NTS, NTF, HVS, and HVF).[7,8] All isolation plates were prepared with sterile water using a MilliQ water purification system (MilliporeSigma) and contained 50 mg/L of cycloheximide and nalidixic acid. Isolation plates ending in "S" contained 31.2g of Instant Ocean sea salt. Plates were incubated at room temperature until the appearance of desired colony morphologies consistent with actinobacteria. Colonies displaying characteristic actinobacterial morphologies (aerial hyphae and substrate mycelia that penetrate the solid agar surface) were subcultured onto either MB (37.4 g Difco™ Marine Broth, 18 g agar, 1 L Milli-Q water) or A1 (15 g agar, 20 g starch, 10 g glucose, 5 g yeast extract, 5 g NZ-amine, 1 g $CaCO_3$, 50 mg nalidixic acid, 50 mg cycloheximide, 31.2 g Instant Ocean, 1 L Milli-Q water) agar plates.

Preparation of prefractions

Marine-derived strains were inoculated from MB or A1 agar plates into 10 mL of modified SYP (mSYP) liquid media (10 g starch, 4 g peptone, 2 g yeast extract, 1 L Milli-Q water, 31.2 g Instant Ocean) in 25 x 150 mm glass culture tubes for 2-3 days at room temperature with shaking at 200 rpm. These small-scale cultures were shaken at 25°C and 200 rpm for a minimum of three days before moving to 60-mL medium-scales. Cultures were stepped up to medium scale by inoculating 3 mL of the small-scale culture into 60 mL of freshly prepared mSYP in wide-mouthed 250-mL Erlenmeyer flasks with small springs. Medium-scale cultures were shaken at 25°C and 200 rpm for 3-7 days. Large-scale cultures were prepared by inoculating 40 mL of medium scale culture into 1 L of freshly prepared mSYP in 2.8-L Fernbach flasks with a large spring and 20 g of pre-washed Amberlite XAD-16 adsorbent resin (DCM, MeOH, and water; Sigma). Large-scale cultures were shaken at 25°C and 200 rpm for 7-10 days depending on colony morphology. At the end of the fermentation period, cells and resin were separated from the culture medium by vacuum filtration using a Whatman® glass microfiber filter and washed with deionized water. Resin and cells from each culture flask were extracted with 250 mL of 1:1 DCM/MeOH. The organic extract was separated from the cells and resin by vacuum filtration and concentrated *in vacuo*.

Crude organic extracts from marine-derived actinobacteria were subjected to solid phase extraction using a Supelco-Discovery C18 cartridge (5 g) and eluted using a MeOH/H_2O step gradient (40 mL; 10% MeOH, 20% MeOH, 40% MeOH, 60% MeOH, 80% MeOH, 100% MeOH, 100% EtOAc) to afford seven fractions. The 10% MeOH fraction was discarded and the remaining six fractions (A – F) concentrated to dryness *in vacuo*. For biological screening, dry prefractions were resolubilized in 1 mL of dimethyl sulfoxide (DMSO) and transferred to deep-well 96-well plates for long-term storage at -70°C.

UPLC-HDMS^E Analysis

DMSO-solubilized prefractions were diluted for UPLC-HDMS^E analysis in 96-well format. Each sample was first diluted 1:40 into DMSO (GC headspace grade, Fisher) to prevent precipitation. Each sample was then diluted 1:25 into 50% MeOH/H_2O (5% DMSO content). Each sample was then diluted 1:10 into 50% MeOH/H_2O for a final dilution of 1:10,000. Samples were then reformatted to 384-well format for analysis.

All measurements were performed with an Acquity UPLC I-Class (Waters) using an HSS T3 C18, 100 mm x 2.1 mm, 1.7 µm column (Waters). Separation of 5 µL of sample was achieved by a gradient of (A) H2O + 0.1% FA to (B) ACN + 0.1% FA at a flow rate of 500 µL/min and 45°C for 7.5 min (5% ACN, 0-0.3 min; 5-90% ACN, 0.3-4.7 min; 90-98% ACN, 4.7-5.5 min; 98% ACN, 5.5-5.8 min; 5% ACN, 5.81-7.5 min). The LC flow was directly infused into the Synapt G2-Si operated in positive ion resolution mode. Analysis was conducted using the $HDMS^E$ mode which was set to alternate between collision energies of 0eV and 30eV every 0.3 sec. The instrument was operated in electrospray mode with 20 µg/mL leucine enkephalin lockspray infusion enabled every 10 sec. Mass spectra were acquired from 50-1500 *m/z* at 2Hz scan rate in continuum mode without lockmass correction.

All samples were analyzed in triplicate for downstream processing. All samples were measured as 384-well plate batches. Measurements for marine-derived extracts were performed over the course of 7 months. Measurements for each 384-well plate were performed within two weeks of each other, with all replicates of a single 384-well plate batch measured before moving on to the next set of samples. The instrument was mass calibrated and collisional-cross section (CCS) calibrated using MajorMix (Waters) between every 384-well set of samples run. The average *m/z* error was never greater than 0.9 ppm for the calibrant signals prior to acquisition.

Data Processing

All raw data files were processed using a customized workflow developed in our laboratory in collaboration with Waters. The initial peak detection and $HDMS^E$ deconvolution software MSeXpress 2.0 was employed to generate peak lists of precursor ions with their associated product ions for each sample. After initial peak detection, a custom Python script was used to remove signals below 100,000 precursor intensity after peak picking and deconvolution. To further ensure the validity of signals across replicates, another custom Python script (replicate comparison) removed any *m/z* feature that was not present in at least two of three analytical replicates within 0.03 Da and 0.05 min buckets. The resulting precursor and product ion lists were converted to the open format mzML. Due to compatibility issues with the final analysis software, these files were further converted to Mascot generic format (MGF) using MSConvert (Proteowizard)[9] with the default settings and no further filtering applied.

Supplementary Note 2. SNAP-MS validation.

Fermentation and extraction

Isolate RL12-121-HVF-C (resulting extract RLUS-2144), isolate RL12-005-AIF-C (resulting extract RLUS-2081), isolate RL12-019-AIF-A (resulting extract RLUS-2079), isolate RL12-115-NTF-A (resulting extract RLUS-2210, and isolate RL12-143-NTF-A (resulting extract RLUS 2090) were inoculated from A1 agar plates into 10 mL of modified SYP (mSYP) liquid media (10 g starch, 4 g peptone, 2 g yeast extract, 1 L Milli-Q water, 31.2 g Instant Ocean) in 25 × 150 mm glass culture tubes for 2 days at room temperature with shaking at 200 rpm before moving to 60-mL medium-scale cultures. Cultures were stepped up to medium-scale by inoculating 3 mL of the small-scale culture into 60 mL of freshly prepared mSYP in wide-mouthed 250-mL Erlenmeyer flasks with small springs. Medium-scale cultures were shaken at 25°C and 200 rpm for 3 days. Large-scale cultures were prepared by inoculating 40 mL of medium-scale culture into 1 L of freshly prepared mSYP in 2.8-L Fernbach flasks with a large spring and 20 g of pre-washed Amberlite HP-7 adsorbent resin (DCM, MeOH, and water; Sigma). Large-scale cultures were shaken at 25°C and 200 rpm for 7days. At the end of the fermentation period, cells and resin were separated from the culture medium by vacuum filtration using a Whatman® glass microfiber filter and washed with deionized water. Resin and cells from each culture flask were extracted with 250 mL of 1:1 DCM/MeOH. The organic extract was separated from the cells and resin by vacuum filtration and concentrated in vacuo.

Fractionation

Crude organic extracts were subjected to solid phase extraction using a Teledyne ISCO CombiFlash C18 cartridge (5 g) and eluted using a MeOH/H_2O step gradient at 20 mL/min (40 mL; 10% MeOH, 20% MeOH, 40% MeOH, 60% MeOH, 80% MeOH, 100% MeOH, 100% EtOAc) to afford seven fractions. The 10% MeOH fraction was discarded and the remaining six (fractions A – F) concentrated to dryness in vacuo.

An aliquot (12 mg) of RLUS-2081B and C (40 and 60 % MeOH respectively) were combined before being subjected to RP-HPLC on a Phenomenex Synergi Fusion-RP C18 (10 x 250 mm, 10 µm) column. A gradient of acetonitrile and H_2O, both with 0.02% formic acid was used to purify desferrioxamine D2 (1 mg) and E (2.2 mg) (8 ml/min; 5% ACN, 0-2 min; 5-30 % ACN, 2-20 min; 30-100% ACN, 20-20.1 min; 100% ACN 20.1-24 min; initial conditions). All of fraction (153 mg) RLUS-2144D (80% MeOH) was subjected to RP-HPLC (20 ml/min, 62:38, H_20: ACN with 0.02 % formic acid) using an Atlantis T3 OBD (19 x 250 mm, 5 µm) column to give surugamide A (4.6 mg). An aliquot (55.2 mg) of RLUS-2079C (60% MeOH) was subjected to RP-HPLC (1.25 ml/min, 80:20 Acetonitrile: H_2O with 0.02% formic acid) on a Phenomenex Kinetex XB-C18 (250 x 4.6 mm, 5µM) column to give AIF-77-B (0.2 mg). An aliquot (590 mg) of RLUS-2090B, C, and D (40, 60, and 80% MeOH, respectively) was subjected to RP-HPLC (1.25 ml/min, 40:60 Acetronitrile: H_2O with 0.02 % formic acid) on a Phenomenex Kinetex XB-C18 (250 x 4.6 mm, 5µM) column to give a fraction containing a mixture of two isomers (6.0 mg). This mixture was separated using RP-HPLC (1.25 ml/min, 38:62 Acetronitrile: H_2O with 0.02 % formic acid) on a Phenomenex Kinetex XB-C18 (250 x 4.6 mm, 5µM) column to give mycosubtilin D (1.9 mg). An aliquot (4 mg) of RLUS-2210D (80% MeOH) was subjected to RP-HPLC on a Phenomenex Synergi Fusion-RP C18 (10 x 250 mm, 10 µm) column. A gradient of acetonitrile and H2O, both with 0.02% formic acid was used to purify compounds in the nactin family (1.1 mg) (8 ml/min; 50-98% ACN, 0-15 min; 98-100% ACN, 15-18 min).

Supplementary note 3. Comparison of annotation accuracy between SNAP-MS and Network Annotation Propagation (NAP).

The NIH Natural Products Library Round 1 and Round 2 molecular network was reproduced using the GNPS workflow[1], but with no spectral library files selected. (https://gnps.ucsd.edu/ProteoSAFe/status.jsp?task=19cf01cbf1d948df8d80c876776aa3e1) This molecular network was analyzed using the network annotations propagation workflow[10] (https://proteomics2.ucsd.edu/ProteoSAFe/status.jsp?task=162af0f95e844203befd29d41dbc3a2f) for comparison with the results of SNAP-MS. The molecular network was filtered to only include subnetworks with more than three nodes. The annotation success for each node was examined by comparison of the known structure with the consensus ID lists generated by NAP and summarized as percent correct within the top ten, top five, and the top ranked answer. Next, we set out to summarize the annotation success at a subnetwork level. We considered a subnetwork to be correctly annotated if the percentage of nodes correctly annotated in a subnetwork were greater than or equal to a set threshold (33% or 50%). Nodes were considered correctly annotated by NAP if the correct answer was present in the top ten annotations or if the correct answer was present in the candidates with a consensus score ≥0.9. The results of this analysis have been summarized in Supplementary table 3 along with definitions of true positives, false positives, true negatives, and false negatives in this context.

Supplementary Note 4. Cosine scoring with *in silico* MS^2 prediction.

To explore the potential use of *in silico* tools to strengthen compound family prediction a subset of samples from both the NIH DDA dataset as well as a subset of the marine actinomycete DIA data was analyzed. *In silico predictions* were made using CFM-ID 4.0 (CFM-ID (wishartlab.com))[11]. MGF files were then created for each compound using the “Predicted Medium Energy MS/MS Spectrum (20V)” output from CFM-ID for all possible answers that were annotated as the M+H for a given mass from the original GNPS network. MGF files were also gathered from the original files that were uploaded to GNPS. Cosine scoring was then performed using a custom script in Python for the following formula:

$$cosine\ score = \frac{\sum_{i=1}^{n} A_i B_i}{\sqrt{\sum_{i=1}^{n} A_i^2}\sqrt{\sum_{i=1}^{n} B_i^2}}$$

where A and B are the normalized MS feature lists from the .mgf files for spectra A and B.

DDA results

Cosine scoring for the NIH DDA dataset showed moderate results for two of three example sets. Example one (Figure S15A) shows moderate cosine scores (maximum of 0.245) for the correct compound family compared to the other possibilities and would likely have pointed towards the correct of the top two prioritized compound families from SNAP-MS. Example 2 (Figure S15B) showed overall very low cosine scores between predicted compounds and original MGF files (maximum of 0.072). The third example (Figure S15C) is similar to the first example with moderate cosine scores (maximum of 0.323) between the *in silico* predictions and the real data. In this case however MS^2 data prediction would point towards a compound family that was not highlighted as the top SNAP-MS answer but in fact is the correct answer.

DIA results

The cosine scoring results for DIA were generally poor. For five of our six test cases the correct answer could be found among the “top hits” for SNAP-MS, however, the *in silico* comparisons received cosine scores of zero with none of the MS^2 peaks between the DIA data and the *in silico* predictions being the same (Figure S16A-E). For the final example (Figure 16F) cosine scoring was similar to the results obtained using DDA data (maximum of 0.270) where including cosine scoring likely would have pointed the user towards the correct of the top hits from SNAP-MS.

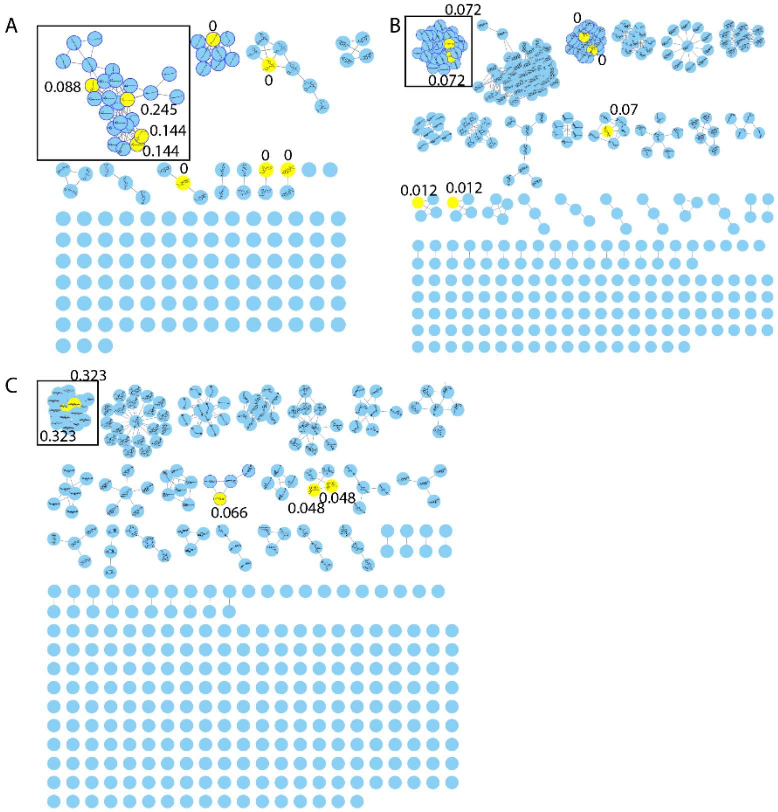

Supplementary Figure 15 | Cosine scoring of NIH DDA data to CFM-ID *in silico* MS^2 data. Examples of using cosine scoring to rank SNAP-MS results for three different GNPS subnetworks (A, B, and C). Cosine score for selected (yellow) nodes shown. The black box is used to designate the correct answer. Nodes with dark blue borders show SNAP-MS top hits.

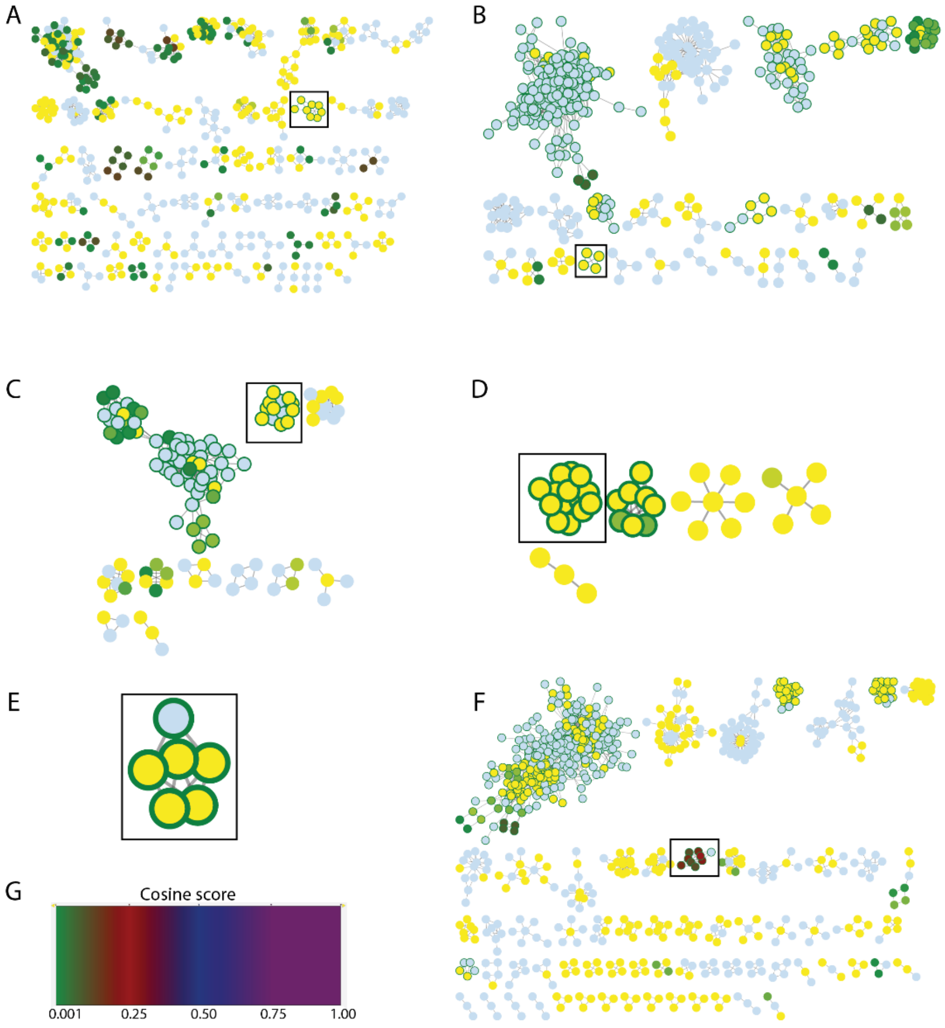

Supplementary Figure 16 | Cosine scoring of marine actinomycete extract DIA data against CFM-ID *in silico* MS^2 data. Examples of using cosine scoring to rank SNAP-MS results for six different GNPS subnetworks (A-F). Cosine score for selected nodes in A-F shown by color scheme G with light gray being features that were not M+H and were not compared, and yellow being a score of 0. The black box is used to designate the correct answer. Nodes with dark green borders show SNAP-MS top hits.

Supplementary References

1. Wang, M. *et al.* Sharing and community curation of mass spectrometry data with Global Natural Products Social Molecular Networking. *Nat. Biotechnol.* **34**, 828–837 (2016).

2. Dührkop, K. *et al.* Systematic classification of unknown metabolites using high-resolution fragmentation mass spectra. *Nat. Biotechnol.* **39**, 462–471 (2021).

3. Tobias, N. J. *et al.* Natural product diversity associated with the nematode symbionts *Photorhabdus* and *Xenorhabdus*. *Nat. Microbiol.* **2**, 1676–1685 (2017).

4. Nguyen, D. D. *et al.* Indexing the *Pseudomonas* specialized metabolome enabled the discovery of poaeamide B and the bananamides. *Nat. Microbiol.* **2**, 16197 (2016).

5. Mudalungu, C. M. *et al.* Noursamycins, chlorinated cyclohexapeptides identified from molecular networking of *Streptomyces noursei* NTR-SR4. *J. Nat. Prod.* **82**, 1478–1486 (2019).

6. Caraballo-Rodríguez, A. M., Dorrestein, P. C. & Pupo, M. T. Molecular inter-kingdom interactions of endophytes isolated from *Lychnophora ericoides*. *Sci. Rep.* **7**, 5373 (2017).

7. Schulze, C. J. *et al.* “Function-first” lead discovery: Mode of action profiling of natural product libraries using image-based screening. *Chem. Biol.* **20**, 285–295 (2013).

8. Wong, W. R., Oliver, A. G. & Linington, R. G. Development of antibiotic activity profile screening for the classification and discovery of natural product antibiotics. *Chem. Biol.* **19**, 1483–1495 (2012).

9. Chambers, M. C. *et al.* A cross-platform toolkit for mass spectrometry and proteomics. *Nat. Biotechnol.* **30**, 918–920 (2012).

10. da Silva, R. R. *et al.* Propagating annotations of molecular networks using in silico fragmentation. *PLOS Comput. Biol.* **14**, e1006089 (2018).

11. Wang, F. *et al.* CFM-ID 4.0: More accurate ESI-MS/MS spectral prediction and compound identification. *Anal. Chem.* **93**, 11692–11700 (2021).
