# Supplementary material for: Annotation of natural product compound families using molecular networking topology and structural similarity fingerprinting
Source: Nat Commun. 2023 Jan 19;14:308. doi: 10.1038/s41467-022-35734-z (PMC9852437; doi:10.1038/s41467-022-35734-z)
Supplement: Supplementary file 3 — Reporting Summary [file 41467_2022_35734_MOESM3_ESM.pdf]

nature portfolio

Corresponding author(s): Linington, RG

Last updated by author(s): Oct 21, 2022

Reporting Summary

Nature Portfolio wishes to improve the reproducibility of the work that we publish. This form provides structure for consistency and transparency in reporting. For further information on Nature Portfolio policies, see our Editorial Policies and the Editorial Policy Checklist.

Statistics

For all statistical analyses, confirm that the following items are present in the figure legend, table legend, main text, or Methods section.

n/a	Confirmed	
☐	☒	The exact sample size (n) for each experimental group/condition, given as a discrete number and unit of measurement
☐	☒	A statement on whether measurements were taken from distinct samples or whether the same sample was measured repeatedly
☒	☐	The statistical test(s) used AND whether they are one- or two-sided<br>*Only common tests should be described solely by name; describe more complex techniques in the Methods section.*
☒	☐	A description of all covariates tested
☒	☐	A description of any assumptions or corrections, such as tests of normality and adjustment for multiple comparisons
☒	☐	A full description of the statistical parameters including central tendency (e.g. means) or other basic estimates (e.g. regression coefficient) AND variation (e.g. standard deviation) or associated estimates of uncertainty (e.g. confidence intervals)
☒	☐	For null hypothesis testing, the test statistic (e.g. F, t, r) with confidence intervals, effect sizes, degrees of freedom and P value noted<br>*Give P values as exact values whenever suitable.*
☒	☐	For Bayesian analysis, information on the choice of priors and Markov chain Monte Carlo settings
☒	☐	For hierarchical and complex designs, identification of the appropriate level for tests and full reporting of outcomes
☒	☐	Estimates of effect sizes (e.g. Cohen's d, Pearson's r), indicating how they were calculated

Our web collection on statistics for biologists contains articles on many of the points above.

Software and code

Policy information about availability of computer code

Data collection	Mass spectrometry (MS) data were acquired on a Waters SYNAPT G2Si mass spectrometer running MassLynx v4.1.
Data analysis	MS data were processed by conversion to mxML format using MSConvert (v3.0.21032-de9bb82e4) from Proteowizard, and replicate compared using MSeXpress (v2.0). The SNAP-MS codebase (v1.0) is available at https://github.com/liningtonlab/snapms. SNAP-MS v1.2.0 is archived at Zenodo under DOI 10.5281/zenodo.7396660. GNPS networks were generated using workflow 18. Network Annotation Propagation results were generated using workflow v1.2.5. Graphs were visualized in Cytoscape v3.7.2. Predicted MS2 spectra in Supplementary Note 4 were generated using CFM-ID 4.0.

For manuscripts utilizing custom algorithms or software that are central to the research but not yet described in published literature, software must be made available to editors and reviewers. We strongly encourage code deposition in a community repository (e.g. GitHub). See the Nature Portfolio guidelines for submitting code & software for further information.

Data

Policy information about availability of data

All manuscripts must include a data availability statement. This statement should provide the following information, where applicable:

- Accession codes, unique identifiers, or web links for publicly available datasets
- A description of any restrictions on data availability
- For clinical datasets or third party data, please ensure that the statement adheres to our policy

The three molecular networks generated in this study have been deposited in the GNPS database are available at https://gnps.ucsd.edu//ProteoSAFe/status.jsp?

task=868a61e685cb401385f1c24bc0edbe62 (NIH 1), https://gnps.ucsd.edu/ProteoSAFe/status.jsp?task=d909a4dccc2747218f9a290d05e7841a (NIH 1 and 2), and https://gnps.ucsd.edu/ProteoSAFe/status.jsp?task=2c39751a78824609a0fdadad989003b6 (actinobacterial library). The two MS reference libraries used in this study are "NIH Natural Products Library Round 1" containing 1,267 spectra available on GNPS (https://gnps-external.ucsd.edu/gnpslibrary/GNPS-NIH-NATURALPRODUCTSLIBRARY.json), and "NIH Natural Products Library Round 2" containing 7,915 spectra and available on GNPS (https://gnps-external.ucsd.edu/gnpslibrary/GNPS-NIH-NATURALPRODUCTSLIBRARY_ROUND2_POSITIVE.json), The actinobacterial mass spectrometry dataset has been deposited in the massIVE database (https://massive.ucsd.edu) under accession code MSV000089680 (https://doi.org/doi:10.25345/C5251FP9X). The Natural Product Atlas database used in this study was v2020_06 and is available from Zenodo (www.zenodo.org) under doi 10.5281/zenodo.6783958 (https://doi.org/10.5281/zenodo.6783958). The COCONUT database used in this study was version January 2022 (https://coconut.naturalproducts.net/download).

Human research participants

Policy information about studies involving human research participants and Sex and Gender in Research.

Reporting on sex and gender	N/A
Population characteristics	N/A
Recruitment	N/A
Ethics oversight	N/A

Note that full information on the approval of the study protocol must also be provided in the manuscript.

Field-specific reporting

Please select the one below that is the best fit for your research. If you are not sure, read the appropriate sections before making your selection.

[x] Life sciences [ ] Behavioural & social sciences [ ] Ecological, evolutionary & environmental sciences

For a reference copy of the document with all sections, see nature.com/documents/nr-reporting-summary-flat.pdf

Life sciences study design

All studies must disclose on these points even when the disclosure is negative.

Sample size	No sample size calculation was performed. 925 extracts were selected as a representative set from our 6,400 member extract library. 925 samples was the largest number of samples that could realistically be analyzed with the available instrument time (925 samples x 3 replicates x 8 minutes duty cycle = 370 hours acquisition (15 days continuous machine time).) Chemical diversity of these samples is unknown so it is not possible to estimate library coverage from this sample set, however this is a much larger NP metabolomics dataset than reported in most other NP studies, illustrating the value of the platform even for large metabolomics datasets.
Data exclusions	MS data were filtered to remove data points with intensities < 50,000 (arbitrary units).
Replication	MS analyses were performed in triplicate. MS features were retained if they were present in at least 2 of 3 replicates. All replicate analyses were included. No replicates were excluded or repeated.
Randomization	Worklists for each MS analysis replicate were separately randomized, so that sample order was random between replicate acquisitions.
Blinding	Compound identities are only determined as the final step in the analysis pipeline once NMR data are acquired, meaning that compound family predictions are blinded to compound identities. No formal sample blinding was performed.

Reporting for specific materials, systems and methods

We require information from authors about some types of materials, experimental systems and methods used in many studies. Here, indicate whether each material, system or method listed is relevant to your study. If you are not sure if a list item applies to your research, read the appropriate section before selecting a response.

Materials & experimental systems

n/a	Involved in the study
☒	☐ Antibodies
☒	☐ Eukaryotic cell lines
☒	☐ Palaeontology and archaeology
☒	☐ Animals and other organisms
☒	☐ Clinical data
☒	☐ Dual use research of concern

Methods

n/a	Involved in the study
☒	☐ ChIP-seq
☒	☐ Flow cytometry
☒	☐ MRI-based neuroimaging
